# Proposal of the Tactile Glove Device

**DOI:** 10.3390/s19225029

**Published:** 2019-11-18

**Authors:** José C. V. S. Junior, Matheus F. Torquato, Daniel H. Noronha, Sérgio N. Silva, Marcelo A. C. Fernandes

**Affiliations:** 1 Laboratory of Machine Learning and Intelligent Instrumentation, Federal University of Rio Grande do Norte, Natal 59078-970, Brazil; cl4udio@gmail.com (J.C.V.S.J.); s.natansilva@gmail.com (S.N.S.); 2 College of Engineering, Swansea University, Swansea, Wales SA2 8PP, UK; m.f.torquato@swansea.ac.uk; 3 Electrical and Computer Engineering, University of British Columbia, Vancouver, BC V6T 1Z4, Canada; holand.daniel@gmail.com; 4 Department of Computer and Automation Engineering, Federal University of Rio Grande do Norte, Natal 59078-970, Brazil

**Keywords:** tactile glove, tactile internet, haptic device, tactile device, wearables

## Abstract

This project aims to develop a tactile glove device and a virtual environment inserted in the context of tactile internet. The tactile glove allows a human operator to interact remotely with objects from a 3D environment through tactile feedback or tactile sensation. In other words, the human operator is able to feel the contour and texture from virtual objects. Applications such as remote diagnostics, games, remote analysis of materials, and others in which objects could be virtualized can be significantly improved using this kind of device. These gloves have been an essential device in all research on the internet next generation called “Tactile Internet”, in which this project is inserted. Unlike the works presented in the literature, the novelty of this work is related to architecture, and tactile devices developed. They are within the 10 ms round trip latency limits required in a tactile internet environment. Details of hardware and software designs of a tactile glove, as well as the virtual environment, are described. Results and comparative analysis about round trip latency time in the tactile internet environment is developed.

## 1. Introduction

Nowadays, the Internet of Things (IoT) enables connecting devices on the Internet in order to increase the applicability of these devices and use the full potential of networks. Research on the network’s area is opening the way to a new generation on the Internet called “Tactile Internet”. The tactile Internet will be able to transform and transmit human sensations through a data network and with the tactile devices; people will be able to receive sensation from a physical and/or virtual object through a machine [1,2,3].

On the tactile internet a bidirectional communication is necessary between the local tactile device (also called master device) and the remote device (also called slave device). The bidirectional communication tries to simulate the physical laws of action and reaction. It is important to emphasize that tactile internet tries to solve a complex problem since the bidirectional communication requires a short latency between 1 and 10 ms for most cases and 100 ms for some cases [4,5,6,7].

Normally, on the tactile system, there are three main elements: the master device, the network, and the slave device. Depending on the type of device that is inserted into the environment, the mode of operation between elements may change. The teleoperated mode is also known as human-to-machine (H2M). In this system, the master device (local device) is controlled by a human operator and the slave device (remote device) is a robotic system [8]. In some cases on machine-to-machine (M2M) systems, the master device can be controlled by a robot where there is not a human in the loop [9,10].

The network is responsible for providing the infrastructure for transferring both haptic information data, kinesthetic, and tactile data between the devices. The communication from the master device to the slave device is called “direct communication” and, it is similar to the telecontrol system. The communication from the slave device to the master device is called “feedback communication” and, it is responsible for transmitting the tactile data that contains the information about sensations (weight, touch, vibration, temperature, and others) or kinesthetic data that contains the information about force [1,2,3].

Tactile internet is an emergent topic and several researchers have been working on this subject. Inside this context, the works with tactile devices are fundamentals because they are main pieces on the tactile system [11,12,13,14,15]. As presented in [1,2,3], the master and slave device must be designed on dedicated hardware with embedded systems, because they need to capture signals from sensors and they need to generate signals to actuators. Another important point is about the algorithms associated to the tactile system, because the embedded system can run complex algorithms related to rotation matrices, matrix transformation, matrix product, non-linear functions, etc. The low processing hardware devices like microcontrollers cannot execute these algorithms in the time restrictions, about 0.15 ms for all processing. There are several kinds of tactile devices such as tactile gloves, robotic arms, exoskeleton hands, kinesthetic haptic device, and others [11,12,16].

Given the diversity of devices, the motivation about this work is to study the challenges contained in the development of tactile internet compatible tactile devices, especially issues related to the round trip latency limit of the system components. The round trip communication between tactile devices and networks must have a latency within limits presented in the literature. In these conditions, this work contributes by presenting a novel embedded design and development of a tactile system that has low latency in communication between devices, respecting the time constraints in the millisecond interval. The tactile system designed has two main devices, a tactile glove, and a virtual environment. In addition, this work presents a comparison with other embedded systems applied to the tactile systems.

The tactile glove created enables the capture of kinematic actions from an operator’s hand and also transmits tactile information to him from virtual objects through tactile feedback. In the virtual environment, in addition to a virtual hand, several virtual objects with different characteristics are created. The operator can control the virtual hand; in the virtual environment, the kinematic equations are implemented according to the characteristics of the hand model. A vital feature of this environment is that the operator wearing the tactile glove can remotely control the virtual hand and receive tactile information that represents the touch on the virtual objects. This tactile information may represent different types of materials and textures depending on the type of object that has been virtualized. Given the characteristics presented, the tactile system could be used on several applications such as telemedicine, remote diagnostics, games, remote analysis of materials, and others in which objects could be virtualized.

## 2. Related Work

In applications involving interactions in virtual environments as well as robot teleoperation applications (human-to-machine), it is necessary to artificially create a sense of touch or force for the operator to be stimulated. From these stimuli, characteristics of objects such as force, texture, weight, and temperature, for example, can be understood by the operator through the received sensation and thus a certain realism can be achieved. To provide this realism, tactile devices are used. As shown in [17], these devices are divided into three categories, being they graspable, touchable, and wearable. The graspable type devices are characterized by being kinesthetic systems, that is, they have force feedback. Touch-sensitive devices are systems that use displays that allow the operator to actively explore the entire surface. Wearable devices are typically characterized by being tactile (cutaneous) systems [17], but it is also possible to find proprioceptive systems [18]. Usually, these devices are mounted on the hands or other parts of the body that transmit sensations directly into the skin. As described in [18], this wearable device is used to convey sensations and for the most part, they are developed in the form of gloves.

However, depending on their architecture, some kinds of gloves can provide both tactile and force feedback. Gloves that transmit force feedback are usually of the exoskeleton type as presented in [19] and [20], these gloves are made up of mechanical parts that are required to provide force feedback. This type of glove is widely used in the rehabilitation and care of people who have some kind of disability [21,22]. Due to the mechanical features aimed at providing the feeling of strength, with this device, it is generally not possible to feel object textures. Tactile gloves are used for this purpose.

Tactile gloves usually differ in the way they detect the movement of the operator’s fingers, arms, and hand. In other words, they may have several degrees of freedom (DoF). Some works have a variety of ways to capture movements. In [23] a camera is used to detect the movement of the fingers, already in [24] a device called LeapMotion is used to capture the movement of the hand and arm. In the same context, the paper presented in [25] shows a rehabilitation system using a virtual reality system with sensory, visual, and auditory feedback. The operator interacts with virtual objects across multiple devices. Arm detection is captured by a Kinect-type human motion detection system and hand movement and finger flexion is captured through a CyberGlove${}^{®}$ II type glove. The defined environment allows very realistic local interaction between the operator and the environment due to the devices used. However, its architecture uses proprietary equipment such as the Kinect, the CyberGlove. The use of these devices may limit the replication of this experiment as they depend on specific hardware.

Another way to capture operator finger and arm positioning is through the use of inertial measurement unit (IMU) sensors as shown in [26,27,28,29]. These sensors allow capturing some kinematics of the hand, including the fingers and forearm. If compared to previous works presented, the use of IMUs sensors can make developing a glove cheaper and easier to replicate. However, the application needs and the development can be complex according to the amount of DOFs to be captured.

When there is no possibility of using sensors to capture the hand movements, the uses of predefined stimuli can assist in the development of applications with tactile actuators. With the glove device presented in [30], it is possible to receive tactile sensations of virtually emulated objects. The glove receives stimuli locally from a tactile information generator server. The stimuli are predefined and sent to the operator without interaction between them. This approach can be useful for validating the types of textures and materials that will be used.

On the other hand, the use of gloves that have only sensitivity sensors can help the way that materials and textures of real objects can be represented virtually. In the works, [31] and [32], two types of high-density tactile detection gloves with 1052 sensitivities elements are proposed. The proposed gloves allow pressure measurement at 1052 points in a human hand. Due to this amount of points, it is possible to detect very small real objects in almost every part of the hand. For the models presented in [31] and [32], the gloves are limited only in capturing the information about the touch of the hand sensors with some type of object, thus differing from the model presented in this work which presents a glove with actuators.

In the context of the tactile internet, artificial skinned humanoids can replace the human operator or even be used as an artificial member of a human operator, enabling exchange information with another type of robot performing the M2M communication. As presented in [33], estimates of contact parameters such as force, soft contact, hardness, texture, and temperature, among other features can be detected by a robot. However, the development of artificial skin can be complex depending on the level of similarity to human skin.

The authors in [34] proposed a low-cost artificial robot skin that could be used to capture tactile touch. With the received data from artificial skin it is necessary to find out the type and the characteristics of the touched material. Some works in the literature discuss how robots can recognize the types of materials and their characteristics. In [35], the authors show how the center of mass of real objects can be obtained. The works [36] and [37] have presented solutions for the recognition of objects through surface textures, it was presented in the methodology that the recognition rate of textures and objects was above 90%. Based on work [38], it is possible to understand how to control the force exerted by a robot’s hands based on the grasp force, as well as to detect the slip of objects.

When a robot starts to perceive the characteristics and properties of an object it may be able to identify it. However, depending on the varying characteristics of the known object (material, texture), there is a possibility that it will not be identified. To enable the identification of variations of object characteristics known by the robot, the authors in [39] and [40] presented algorithm models that aimed solving this problem. The work [41] presents a robot capable of identifying unknown objects by their physical properties (surface texture, stiffness, and thermal conductivity).

Among the works presented, those that focus on machine-to-machine applications ([31,32,34,35,36,37,38]), are more focused on the development of devices with sensors for texture detection and recognition. This is different from the proposal of this work, which is focused on the development of a tactile glove with IMU sensors and vibration actuators that are activated when there is some kind of interaction with virtual objects.

In the human-to-machine system line, the architectures presented in the works [27,28,29] allow the tactile glove to handle real robotic systems. However, when there is no physical model, a new architecture must be developed. Another important point is that in these environments, textures and virtual objects cannot be felt.

As can be seen from the works [27,28,29], gloves differ in design and some features. For example, the manner in which the position of the fingers, hand, and arm is captured. Another point is how the glove communicates with the controlled device. It is also important to emphasize that in none of these works is it possible to perform a glove interaction with virtual objects, only the work [30] allows the reception of already predefined stimuli. Therefore, in this work, a complete environment is proposed so that operators with the tactile glove can interact and feel textures remotely from a virtual environment. Unlike the work presented in this proposal, a complete specification of the environment will be provided, both the glove design and the electronics, as well as the virtual model.

## 3. System Architecture

**Figure 1:**
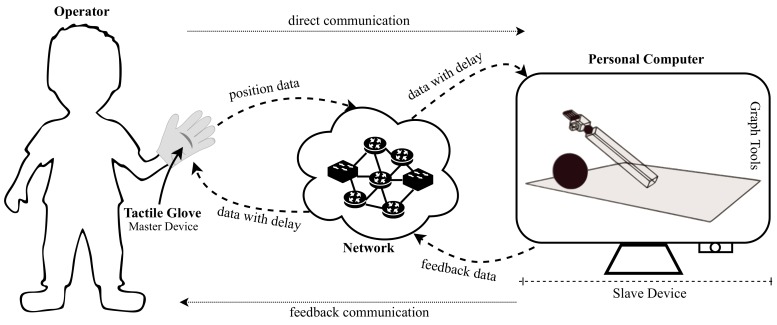
High-level block diagram of the human-to-machine tactile system.

The high level block diagram presented in Figure 1 presents an overview of the envisaged scheme which represents the tactile system. The scheme basically has a local device (known as master) and a remote device (known as slave) that communicate over the internet through a bidirectional data communication network. The master device is a tactile glove which is controlled by an operator and the slave device is a personal computer showing a virtual robotic manipulator.

As can be seen in Figure 1, the operator wearing the tactile glove can remotely control a robotic manipulator to do the desired task. The initial step is identified as the movement which the operator performs when wearing the glove. These movements are detected by the sensors present on the glove and sent to the computer to control the virtual robotic manipulator. The second step is identified as the data communication network between the master and slave devices. This network is connected to the Internet is usually composed of transmitters, routers, switches, and other communication components. The subsequent step is identified as the steps performed by the personal computer so that the virtual robotic manipulator performs the movements sent by operator. In this stage, the collision and feedback control are generated so that stimuli are sent to the operator. The final step is identified as the result of the process of operator interaction with the virtual environment. In this step, the feedback signals can be received by the actuators present in the glove to transmit the vibrotactile sensation to the operator.

**Figure 2:**
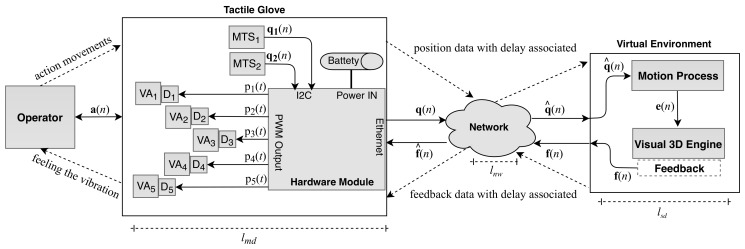
Block diagram of the human-to-machine tactile system architecture.

To better understand the steps presented, Figure 2 shows the general proposed architecture scheme which represents the tactile system. The proposed model is formed by four subsystems called operator (OP), tactile glove (TG), network (NW) and virtual environment (VE). The tactile glove is equipped with sensors and actuators that allow the operator to interact and manipulate objects that are inserted into a virtual environment, aiming to perform some type of task. Data communication between the tactile glove and the virtual environment occurs through the network.

The tactile glove is composed of two motion tracking sensors, called here MTS${}_{1}$ localized on the hand and MTS${}_{2}$ localized on the arm, five vibration actuators localized on the fingers, called VA${}_{i}$, where i=1…5 and a hardware module, five drivers, called D${}_{i}$, where i=1…5 and battery. The network provides an infrastructure to transmit signals from the operator to the virtual environment and feedback signals in the reverse direction. The virtual environment is composed by a PC running a virtual engine 3D.

When an operator is using the tactile glove he can begin to interact with the virtual environment. As shown in Figure 2, the signal a(n) represents the kinematics movement performed by the operator. When the operator carries out some type of kinematic movement, the *j*-th MTS modules present on glove hardware compute the resulting position of operator movement in terms of quaternions at each *n*-th instant, and send this information through the discrete signal by a vector $q_{j}\left(n\right)$ expressed as

(1) $$\begin{array}{cc} q_{j}\left(n\right)= & \left[\begin{array}{c} qw_{j}\left(n\right)\\ qx_{j}\left(n\right)\\ qy_{j}\left(n\right)\\ qz_{j}\left(n\right) \end{array}\right]\\ = & \left[\begin{array}{c} \cos\left(\frac{\theta (n)}{2}\right)\\ vx_{j}\left(n\right)\sin\left(\frac{\theta (n)}{2}\right)\\ vy_{j}\left(n\right)\sin\left(\frac{\theta (n)}{2}\right)\\ vz_{j}\left(n\right)\sin\left(\frac{\theta (n)}{2}\right) \end{array}\right] \end{array}$$

where $\left[\theta ,vx,vy,vz\right]$ are the four parameters that define the quaternion. θ(n) is the angle of rotation and vx, vy, and vz represent the axis of rotation.

As shown in Figure 2, after the hardware module receives the $q_{1}\left(n\right)$ and $q_{2}\left(n\right)$ signals through the I2C communication protocol it creates a new discrete signal by a vector to be sent to the network. The newly created signal q(n) is expressed as

(2) $$q\left(n\right)=[q_{1}\left(n\right),q_{2}\left(n\right)]$$

where $q_{1}\left(n\right)$ is the quaternion information about module MTS${}_{1}$ and $q_{2}\left(n\right)$ is the quaternion information about module MTS${}_{2}$.

When the signal q(n) sent by the tactile glove is transmitted and propagated through the network to the virtual environment, this signal can have some type of disturbance. So when the network receives the signal, a delay here called $d^{f}$ is considered, resulting in a new signal $\hat{q}\left(n\right)$ which is expressed as

(3) $$\hat{q}\left(n\right)=\left[q_{1}\left(n-d^{f}\right),q_{2}\left(n-d^{f}\right)\right]$$

where $q_{1}\left(n-d^{f}\right)$ and $q_{2}\left(n-d^{f}\right)$ are the data transmitted by the network with a delay $d^{f}$ at the *n*-th instant of time.

As soon as the $\hat{q}\left(n\right)$ signal arrives in the virtual environment, it is directed to the motion process module which is responsible for processing information related to the movements in the virtual environment.

Then, with the quaternion information received through the $\hat{q}\left(n\right)$ signal, it is possible to determine the angular vector of rotation, also called the Euler angles associated with the tactile glove. Thus, the signals containing the quaternions $q_{1}\left(n\right)$ and $q_{2}\left(n\right)$ are transformed into Euler angles so that the positioning of the hand (MTS${}_{1}$) when the arm (MTS${}_{2}$) is determined. This process is performed every *n*-th instant and sent to the visual 3D engine module via signal discrete $e_{j}\left(n\right)$ which is expressed as

(4) $$\begin{array}{cc} e_{j}\left(n\right)= & \left[\begin{array}{c} \phi_{j}\left(n\right)\\ \theta_{j}\left(n\right)\\ \psi_{j}\left(n\right) \end{array}\right]\\ = & \left[\begin{array}{l} \;\;\;\arctan\left(\frac{2(qw_{j}\left(n\right)qx_{j}\left(n\right)+qy_{j}\left(n\right)qz_{j}\left(n\right))}{qw_{j}{\left(n\right)}^{2}-qx_{j}{\left(n\right)}^{2}-qy_{j}{\left(n\right)}^{2}+qz_{j}{\left(n\right)}^{2}}\right)\\ -\arcsin\left(2(qx_{j}\left(n\right)qz_{j}\left(n\right)-qw_{j}\left(n\right)qy_{j}\left(n\right))\right)\\ \;\;\;\arctan\left(\frac{2(qw_{j}\left(n\right)qz_{j}\left(n\right)+qx_{j}\left(n\right)qy_{j}\left(n\right))}{qw_{j}{\left(n\right)}^{2}+qx_{j}{\left(n\right)}^{2}-qy_{j}{\left(n\right)}^{2}-qz_{j}{\left(n\right)}^{2}}\right) \end{array}\right] \end{array}$$

where j=1…2 represents the MTS${}_{j}$ values, $\phi_{j}\left(n\right)$, $\theta_{j}\left(n\right)$ and $\psi_{j}\left(n\right)$ are called the yaw, pitch, and roll, respectively.

At the moment the $e_{j}\left(n\right)$ signals are received by the visual 3D engine, it is possible to calculate the current glove position in space, expressed by vector $s_{j}\left(n\right)=\left[s_{j}^{x}\left(n\right),s_{j}^{y}\left(n\right),s_{j}^{z}\left(n\right)\right]$ for hand and arm through kinematic calculations or through calculations using rotational matrices. Thus, after performing these calculations it is possible to display the positioning of the tactile glove in a virtual way. To do this, the application created in visual 3D engine that implements the virtual model of the manipulator performs the positioning of the hand and the arm every *n*-th instant.

After the virtual manipulator begins to move, it can find some virtual objects in the way. Virtual objects are also created in the visual 3D engine; they can be made with different types of materials and textures. When the operator virtually touches objects, the collision detection routines are triggered to generate some kind of stimulus. The touch sensation is sent from the virtual environment to the operator via tactile feedback.

When the virtual tactile glove moves in the environment at every *n*-th instant, the equation responsible for detecting the collision is performed, it is expressed as

(5) $$c\left(n\right)=\frac{\left(N_{a}s_{j}^{x}\left(n\right)+N_{b}s_{j}^{y}\left(n\right)+N_{c}s_{j}^{z}\left(n\right)\right)-\left(N_{a}x0+N_{b}y0+N_{c}z0\right)}{\sqrt{N_{a}^{2}+N_{b}^{2}+N_{c}^{2}}}$$

where N = [$N_{a}$, $N_{b}$, $N_{c}$] is a normal vector and x0, y0, and z0 is the position of the virtual object.

After the collision routines are executed, if a touch is detected then the routine responsible for generating the tactile feedback is triggered. The tactile feedback routine is based on the spring-damper force model as presented in [42]. The tactile feedback for each *i*-th VA${}_{i}$ is obtained by the equation expressed as

(6) $$f_{i}\left(n\right)=k_{i}c\left(n\right)$$

where i=1…5 and $k_{i}$ is the *i*-th spring constant.

Then the feedback information (about sensation) is sent to the master device through the discrete signal of a vector that can be expressed as

(7) $$f\left(n\right)=\left[f_{1}\left(n\right),\ldots ,f_{5}\left(n\right)\right]$$

where $f_{i}\left(n\right)$ is the signal associated of the *i*-th finger at the *n*-th instant of time. The signal $f_{i}\left(n\right)$ can be a value between zero and 100. These values can be changed according to the type of force exerted on the virtual object.

As shown in Figure 2, the f(n) signal that is associated with feedback information is sent from virtual environment to tactile glove through the network. As previously stated, the signals transmitted by the network may suffer perturbations, so a new discrete signal by a vector $\hat{f}\left(n\right)$ is created being expressed as

(8) $$\hat{f}\left(n\right)=\left[f_{1}\left(n-d^{b}\right),\ldots ,f_{5}\left(n-d^{b}\right)\right]$$

where $f_{i}\left(n-d^{b}\right)$ is the data transmitted by the network with a delay $d^{b}$ at the *n*-th instant of time.

Thereafter, the hardware module on the tactile glove receives the $\hat{f}\left(n\right)$ signal and calls the routines responsible for providing feedback to the operator. The technique consists in varying the working time for each *i*-th VA${}_{i}$ actuator, where it increases according to the pressure exerted on the virtual object. Each vibration actuator, VA${}_{i}$, was governed by a driver, $D_{i}$, using a pulse width modulation (PWM) signal, $p_{i}\left(t\right)$, expressed as

(9) $$p_{i}\left(t\right)=\left\{\begin{array}{cc} a & \;\mathrm{if}\;\frac{f_{i}\left(n\right)}{100}>c\left(t\right)\\ \;\\ 0 & \;\mathrm{if}\;\frac{f_{i}\left(n\right)}{100}\leq c\left(t\right) \end{array}\right.$$

where *a* is the amplitude of the signal, $f_{i}\left(n\right)$ is the pulse width, which varied from 0 to 100%, and c(t) is a sawtooth signal with amplitude 1 and frequency $f_{PWM}$. The driver, $D_{i}$, regulated the voltage at the terminals of the VAs according to

(10) $$v_{i}\left(t\right)=f_{i}\left(n\right)v_{a}^{max}\left(t\right)$$

where $v_{a}^{max}\left(t\right)$ is the maximum voltage at the terminals of the each *i*-th actuator VA${}_{i}$.

**Figure 3:**
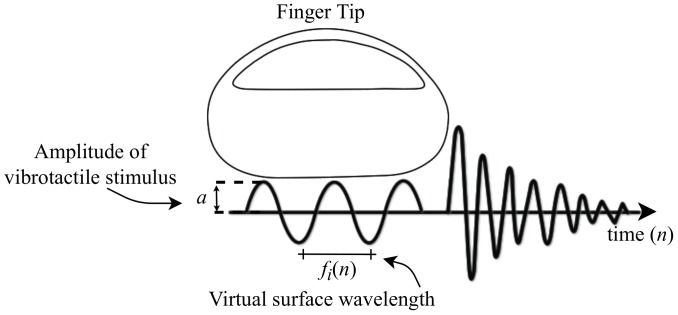
The vibrotactile stimulus for sensations.

At the end of the process, with PWM techniques it is possible to change the vibrations so that the glove produces tactile stimulation through the actuators, as can be seen in Figure 3. The wavelength of the virtual surface can be modified at each instant of time so that the operator feels vibrations that inform them about the object they are manipulating.

## 4. Description of the Design

This section includes hardware and software design of the system architecture shown in Figure 2. The source codes as well as detailed information about the hardware implementation are available at https://github.com/danielholanda/Tactile-Glove.

### 4.1. The Tactile Glove

#### 4.1.1. Embedded System

The embedded system was developed for Intel Galileo 2nd Generation. This microcontroller board is based on the Intel® Quark SoC X1000 application processor of the 32-bit Pentium class.

In the glove device each *j*-th MTS is the MPU-6050, where each IMU contains a digital motion processor (DMP) which fuses the accelerometer and gyroscope data together (Six-Axis Gyro + Accelerometer). The MTS-1 is localized on the wrist and the MTS-2 is localized on the center of the hand. With the two MTS it is possible to obtain the spatial localization on the hand and the forearm at the *n*-th instant of the time.

**Figure 4:**
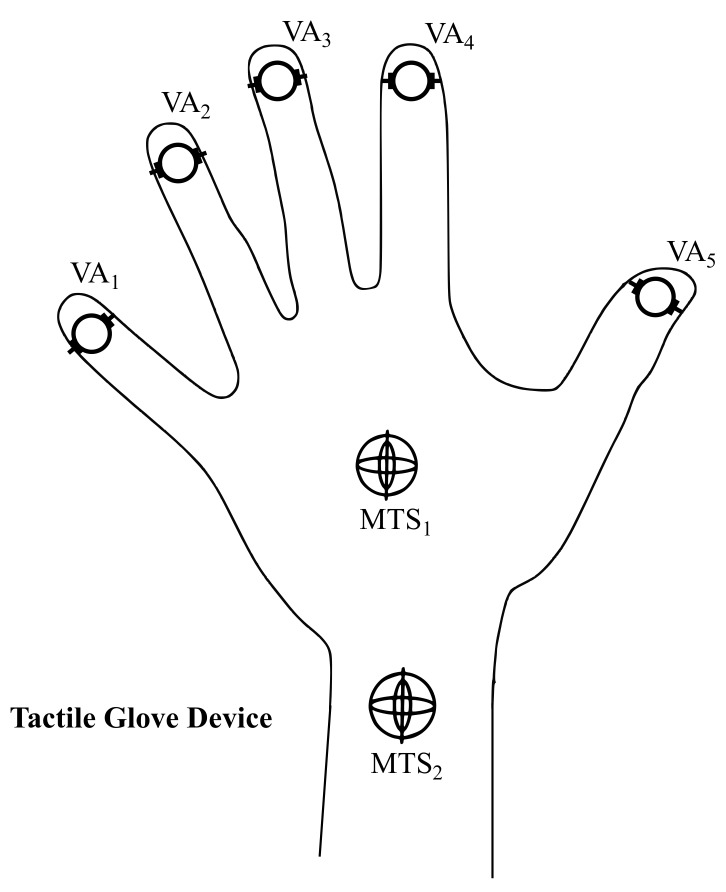
Position of sensors’ motion tracking sensors (MTSs) and actuators’ vibration actuators (VAs) in tactile glove.

In addition, there are the five vibration actuators; VAs provides tactile feedback to the glove. To provide the sensation of vibration, the eccentric rotating mass motor (ERM) is used. Each *j*-th is an ERM and vibrates when a DC voltage is applied across it. The VA${}_{1}$ is located on the lower tip of the thumb, the VA${}_{2}$ is located on the index, the VA${}_{3}$ is located on the middle, the VA${}_{4}$ is located on the ring, and the VA${}_{5}$ is located on the pinky (see Figure 4).

**Figure 5:**
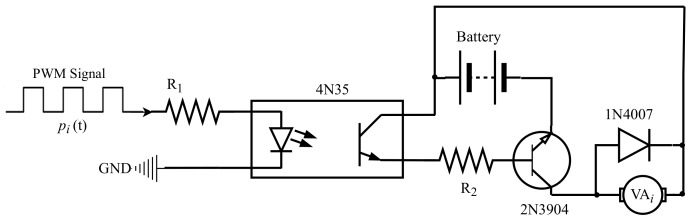
The driver circuit associated to each vibration actuator, VA${}_{i}$.

As shown in the Figure 2, between hardware module and each *i*-th VA${}_{i}$ there is a driver circuit D${}_{i}$. The driver circuit associated to each VA${}_{i}$ is shown in Figure 5.

This circuit is composed by two resistors, $R_{1}$ and $R_{2}$, with values 390Ω and 4.7Ω respectively, one optoacoplador 4N35, one transistor NPN 2N3904, one rectifier diode 1N4007, and a battery.

#### 4.1.2. Software

The Hardware module is running Linux Yocto and there is an embedded application in C++ called here MasterApp. Two important libraries are used to carry out this process. The first one is used to provide I2C communication between MasterApp and each *j*-th MTS (shown in Figure 2), for that was used i2cdevlib. The second library is used to control GPIOs and for that is used a low-level C/C++ library called MRAA. This library provides bindings in a few programming languages to I/O interface in several hardware platforms. Using this library it is possible to create a code that is compatible with various hardware platforms. That is, its use is not tied to a specific hardware.

The steps processed by the MasterApp are presented in Algorithm 1 and are described in detail below.

In the first step (line 1 of Algorithm 1), the MasterApp initializes the variables and libs. After that, the MasterApp stays in a loop until the simulation is stopped. When the simulation is in progress, it is verified if the I2C connections to the MTS are working properly. If everything is working, the steps presented in lines 4, 5, and 6 are performed.

In the steps shown in lines 4 and 5, the MasterApp captures quaternion information from each *j*-th MTS through I2C communication. Being that the variable $q_{1}\left(n\right)$ receives the information from address 0X68 and $q_{2}\left(n\right)$ from address 0X69.

As described in Equation (1) each signal $q_{j}\left(n\right)$ is composed of four pieces of information. Thus, a new variable q(n) (as presented in line 6 and according to Equation (2)) containing a packet of information composed by $q_{1}\left(n\right)$ and $q_{2}\left(n\right)$ is created to be sent to SlaveApp through a TCP socket. If everything is doing right, the variable q(n) is sent to SlaveApp as described in line 9 of Algorithm 1.

After these steps the information about feedback $\hat{f}\left(n\right)$ can be received, according to the step of line 11. With the values obtained by signal $\hat{f}\left(n\right)$, it is possible to generate the sensation of tactile feedback through the PWM modulation. For that the step as shown in line 12 must be processed according to Equation (9). After that, the MasterApp performs the next loop.
**Algorithm 1:** MasterApp (Glove Device Algorithm)1 initialization;
2 **while**
*simulationIsRunning*
**do**
((
3 **if**
*isDMPReady*
**then**((
4  $q_{1}\left(n\right)\leftarrow getQuaternionFromI2C\left(0x68\right)$;
5  $q_{2}\left(n\right)\leftarrow getQuaternionFromI2C\left(0x69\right)$;
6  $q\left(n\right)\leftarrow generateQuaternionPacket\left(q_{1}\left(n\right),q_{2}\left(n\right)\right)$;
7 **end**(
8 **if**
*hasIMUData*
**then**((
9  sendQuaternionToSlave(q(n));
10 **end**(
11 $\hat{f}\left(n\right)\leftarrow readFeedbackDataFromNetwork\left(\right)$;
12 $p\left(t\right)\leftarrow generateFeedbackSensationWithPWM\left(\hat{f}\left(n\right)\right)$;
13 **end**(


### 4.2. The Network

The network is the communication medium used to transmit the tactile glove’s actuation signals to the virtual environment as well as the feedback signals sent from the virtual environment to the glove. Applications that are in the context of the tactile Internet often require a network environment that has very low latency. However, since the main purpose of this work is the development of the tactile glove architecture and not of the network environment, it is assumed that the use of a local network routing device satisfies this requirement. Therefore, an Askey RTF3505VW-N2 router model was used to enable the tactile glove and the virtual environment to communicate over a wired LAN network with connection on the internet.

### 4.3. The Virtual Environment

The virtual robotic hand was modeled using the software Processing 3. Processing is a flexible software with an easy language for development of virtual environments. The steps processed by the SlaveApp are presented in Algorithm 2 and are described in detail below.
**Algorithm 2:** SlaveApp (Virtual Environment Algorithm)1 initialization;
2 **while**
*simulationIsRunning*
**do**
((
3  $\hat{q}\left(n\right)\leftarrow readQuaternionFromNetwork\left(\right)$;
4  $e\left(n\right)\leftarrow getEulerAngles(\hat{q}\left(n\right))$;
5  moveVirtualArmHand(e(n));
6  f(n)←detectCollision();
7  sendFeedbackToMaster(f(n));
8 **end**(


In the first step (line 1 of Algorithm 2), the SlaveApp initializes the variables and libs. After that, the SlaveApp stays in a loop until the simulation is stopped. When the simulation is in progress, the SlaveApp receives $\hat{q}\left(n\right)$ as quaternion packet information from MasterApp through TCP socket, as shown in step in line 3. In the next step the variable e(n) is obtained as shown in line 4 after transformate quaternion packet in euler angles. Posteriorly moves the virtual robotic hand.

While the hand is moving around the environment, any collision on objects created in the virtual environment can be detected the step shown in line 6. Finally the data f(n) about feedback is sent to MasterApp. After that, the SlaveApp perform the next loop.

## 5. Results

**Figure 6:**
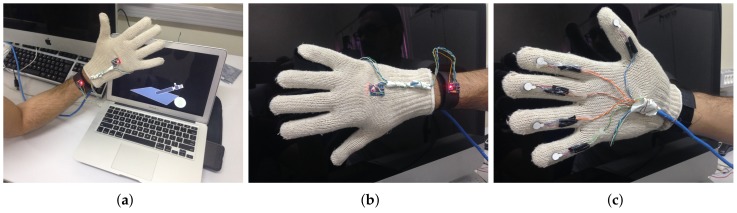
The final result of the design of the proposed glove. (**a**) Tactile glove and slave device (PC with virtual robotic arm). (**b**) Position of inertial measurement units (IMUs) (motion tracking device) in the tactile glove. (**c**) Position of all fingers’ actuators (five vibration actuators) in the tactile glove.

The final result of the proposed glove can be seen in illustrations presented in Figure 6. In the illustration of Figure 6a, it is possible to observe the tactile glove (master device) controlling the virtual environment (slave device). In the illustration Figure 6b are the sensors MTS${}_{1}$ and MTS${}_{2}$. Finally, in the illustration of Figure 6c, it is possible to observe the five vibrotatile actuators’ VAs.

**Figure 7:**
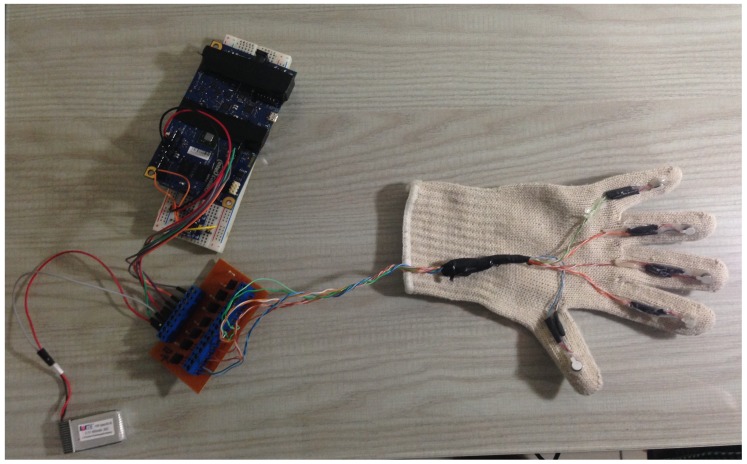
Final version of the hardware.

In Figure 7 the developed hardware used for controlling the tactile glove is presented. It contains the Galileo Gen2 board, the drivers, and the battery.

### 5.1. Round Trip Delay and Component Latencies

**Figure 8:**
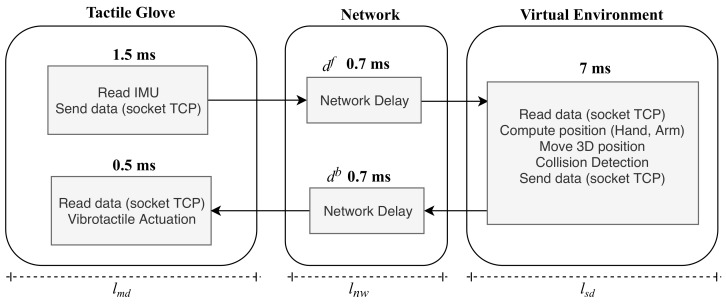
Component latencies. Round trip latency is 10.4 ms.

Based on this, a brief analysis of the delay of the modules involved in this work is carried out. Figure 8 provides an overview of the developed environment. It is possible to observe five steps that are performed so that the entire cycle of interaction between the tactile glove and the virtual environment is realized.

The first step is related to the delay spent by the glove device; it involves the process of reading the IMUs (MTS${}_{1}$ and MTS${}_{2}$) and sending the information through the TCP socket. These processes take 1.5 ms to be finalized.

The delay related to data transmission over the network is defined as $d^{f}$ for when the signals are transmitted from the master device to the slave and $d^{b}$ when the signals are transmitted in the reverse path. In the architecture used, the values for $d^{f}$ and $d^{b}$ are 0.7 ms and 0.7 ms respectively. Thus, the total latency spent by the network $l_{nw}$ is given by the delays $d^{f}$ and $d^{b}$, which has a total of 1.4 ms.

The step which composes the virtual environment involves the process of calculating the position of the hand—rendering of the positioning of the hand in the 3d environment—and the collision process, which also involves feedback. The total latency for this step is given by $l_{sd}$, which has a value of 7 ms.

The feedback sent by the virtual environment goes through the network again with the delay already shown. Finally, the hardware present in the tactile glove receives the feedback signals through the socket and generates the PWM signals for the vibrator actuators; this process generates a delay of 0.5 ms.

The total latency of the system is given by the sum of processing time spent on the glove added to the total transmission time plus the total processing time spent by the virtual environment. Thus, the total latency of the system was calculated by the equation expressed as

(11) $$l_{total}=l_{md}+l_{nw}+l_{sd}$$

The latency obtained from the tactile glove $l_{md}$ and the latency obtained from the virtual environment $l_{md}$ is high due to the hardware model used. Another limiting point is the transmission rates between the components due to the communication protocols that were used.

The round trip latency of the environment was about 10.4 ms. Thus, with this obtained value it is possible to conclude that this application is within the requirements necessary to be used in tactile internet applications [4,5,6].

### 5.2. Related Works Comparison

Table 1 shows a comparison of the related works. The first column presents the related works. The second, the hardware model that is integrated into each project. The third and fourth columns are related to the processor type used in the glove hardware, where the third shows information about MIPS/MHz/Core processing efficiency and the fourth, the number of processor bits. The last two columns show the number of sensors and actuators used on the sleeve respectively.

As can be seen from Table 1, there were variations in the type of hardware used in developing the gloves. The work presented in [27] used an FPGA board, the works [26,29,30] used microcontrollers with 16, 8, and 8 bits respectively. Only the work [28] used a 32-bit microprocessor equivalent to what is being used in the proposal presented in this work. It can be noticed that all the related works presented in Table 1 used some type of sensors. The IMU was the one chosen in most of the projects and it is used to capture finger, hand, forearm, and arm movements. Unlike other works, the authors in [29,30] used flexible resistive sensors to capture finger position. Regarding the actuators, only the work [26] did not use any. All others used vibrating actuators, differing only in the amount used.

Table 2 shows the comparison of other characteristics in relation to the same works that were presented in Table 1. In Table 2, The first column identifies each related work. Subsequently, the second column shows in which locations the sensors and actuators shown in Table 1 were allocated. The third column represents information about the use of tactile feedback. The fourth column is related to the type of communication that was used to communicate between the glove and the device. Finally, the last two columns present whether the proposed environment enables communication through the internet and if the developed architecture allows the glove to communicate with any virtual environment.

As can be observed in Table 2 only the glove proposed in this work has a TCP communication interface with the internet without the need for extra devices. The works [26,29,30] only allow local communication via Bluetooth with the slave device. In work [27], even though the glove has wifi connectivity, the environment does not provide an internet connection. The work [28] allows an internet connection, however, the glove is dependent on a UART connection with a personal computer.

An important point in the proposal of this work is the interaction of the glove with virtual objects which allows the identification of different textures. As shown in Table 2, only work [30] has a virtual environment, but the proposed environment does not allow communication over the internet and interaction is limited only to the reception of pre-defined sensations.

Table 3 presents the round trip and speedup measurement results of the related works. Among the works presented in the previous tables, only works [26,28,29] present latency results of the developed environment. These works are listed in the first column of Table 3, in the second column, it is possible to observe the round trip latency. Finally, the last column presents the speedup obtained in relation to the proposal presented in this work with the references.

The work [28] shows a round trip latency of 85 ms. Although the authors use a 32-bit microprocessor, high latency may be caused by the type of protocol used for communication between components. In work [29] the results indicate that the main loop of the application is executed with a frequency of 25 Hz. This value is equivalent to a 40 ms round trip latency.

The round trip latency values of the works [28] and [29] are higher than the result obtained by the work here presented. As can be seen from Table 3, this work has a speedup of 8.17 times faster than [28] and 3.85 times faster than [29].

At the moment, the main limitation of the prototype presented in this work is related to the number of existing actuators. However, this amount can be expanded. Additionally, the presented prototype can be improved by using dedicated hardware to speed up data processing. As a result, information execution time may decrease, and the round trip latency can be shorter.

## 6. Conclusions

This work presents a proposal for the implementation of a tactile glove and a virtual environment inserted in a tactile internet environment. The model gives the operator direct contact with the virtual objects, giving the impression that there is physical touch. With this, the operator can perceive what type of product or material is being touched, in addition to being able to feel different types of textures. The proposed model differs from the works presented in the literature since the interaction between the real and the virtual world is independent of the location of the master and slave devices. The glove and the virtual environment may be in the same place or even geographically distant because in the tactile Internet environment this limitation is solved. Full details of the architecture and implementation of the tactile glove as well as the virtual environment are provided with the aim of contributing to the development of new applications for the tactile Internet. The practical results obtained confirm that the model works as expected and demonstrate the viability of the application in tactile Internet environments. 

## Figures and Tables

**Table 1:** Comparison of the hardware, sensors, and actuators used in this work with other works.

Reference	Glove Hardware	MIPS/MHz/Core	Processor Bits	Sensors	Actuators
[26]	MSP430F5438A	-	16	18 IMUs	No
[27]	FPGA DE0-nano	-	-	11 IMUs	14 Vibrotactile
[28]	Cypress PSoC 5LP	1.25	32	5 IMUs	2 Vibrotactile
[29]	ATmega32U4	-	8	10 Flex + 1 IMU	5 Vibrotactile
[30]	PIC	-	8	5 Flex	5 Vibrotactile
This work	Galileo Gen2	1.25	32	2 IMUs	5 Vibrotactile

**Table 2:** Comparison of our proposed glove and other gloves.

Reference	Mov. Detection	Feedback	Communication	Internet	Virtual Env.
[26]	Finger + Hand + Forearm	No	Bluetooth	No	No
[27]	Finger	Yes	UART+Wifi	No	No
[28]	Finger + Hand + Forearm + Arm	Yes	UART + PC	Yes	No
[29]	Finger + Hand	Yes	Bluetooth	No	No
[30]	Finger	Yes	Bluetooth	No	Yes
This work	Hand+Forearm	Yes	TCP	Yes	Yes

**Table 3:** Round trip latency and speedup measurement results.

Reference	Round Trip Latency	Speedup
This work	10.4 ms	-
[28]	85 ms	8.17
[29]	40 ms	3.85
